# Usability and Effectiveness of Immersive Virtual Grocery Shopping for Assessing Cognitive Fatigue in Healthy Controls: Protocol for a Randomized Controlled Trial

**DOI:** 10.2196/28073

**Published:** 2021-08-04

**Authors:** James A Holdnack, Patricia Flatley Brennan

**Affiliations:** 1 National Institute of Nursing Research National Institutes of Health Bethesda, MD United States

**Keywords:** cognitive fatigue, immersive VR, user experience, virtual grocery shopping, instrumental activity of daily living

## Abstract

**Background:**

Cognitive fatigue (CF) is a human response to stimulation and stress and is a common comorbidity in many medical conditions that can result in serious consequences; however, studying CF under controlled conditions is difficult. Immersive virtual reality provides an experimental environment that enables the precise measurement of the response of an individual to complex stimuli in a controlled environment.

**Objective:**

We aim to examine the development of an immersive virtual shopping experience to measure subjective and objective indicators of CF induced by instrumental activities of daily living.

**Methods:**

We will recruit 84 healthy participants (aged 18-75 years) for a 2-phase study. Phase 1 is a user experience study for testing the software functionality, user interface, and realism of the virtual shopping environment. Phase 2 uses a 3-arm randomized controlled trial to determine the effect that the immersive environment has on fatigue. Participants will be randomized into 1 of 3 conditions exploring fatigue response during a typical human activity (grocery shopping). The level of cognitive and emotional challenges will change during each activity. The primary outcome of phase 1 is the experience of user interface difficulties. The primary outcome of phase 2 is self-reported CF. The core secondary phase 2 outcomes include subjective cognitive load, change in task performance behavior, and eye tracking. Phase 2 uses within-subject repeated measures analysis of variance to compare pre- and postfatigue measures under 3 conditions (control, cognitive challenge, and emotional challenge).

**Results:**

This study was approved by the scientific review committee of the National Institute of Nursing Research and was identified as an exempt study by the institutional review board of the National Institutes of Health. Data collection will begin in spring 2021.

**Conclusions:**

Immersive virtual reality may be a useful research platform for simulating the induction of CF associated with the cognitive and emotional challenges of instrumental activities of daily living.

**Trial Registration:**

ClinicalTrials.gov NCT04883359; http://clinicaltrials.gov/ct2/show/NCT04883359

**International Registered Report Identifier (IRRID):**

PRR1-10.2196/28073

## Introduction

### Background

The application of digital technologies to improve the monitoring and treatment of chronic clinical conditions is an emerging field in medical research and practice. At the most basic level, the maintenance of and nearly instantaneous access to medical records facilitates tracking and coordination of care among providers is an example of how digital technologies have directly influenced the practice of medicine. The steady increase in apps and digital devices developed to track health-related behaviors and monitor physiological data is a testament to the interest and potential powerful role that technology will play in the future of medicine. These tools may become most useful for aiding health care in the gaps between formal treatment (eg, hospital, clinic, and doctor visit) and day-to-day living in extended or chronic conditions. For example, individuals with chronic medical conditions often experience significant symptoms of cognitive fatigue (CF); however, it is a challenge for clinicians to evaluate the impact of this symptom on daily activities. Technological solutions potentially provide greater insight into the impact of symptomatology on the quality of life. Researchers and clinicians alike have a profound interest in technology and its current and future role in health care delivery.

### Immersive Virtual Reality

Immersive virtual reality (VR) technology has been increasingly used by researchers in many fields as a tool to observe and measure the responses of individuals to complex stimuli in a controlled environment [1-3]. Auditory and visual stimuli induce the sense that they are in a space different from where their physical body is located. Usual tasks (locomotion, pointing, and grasping) are accomplished in a modified manner using ancillary equipment (eg, hand controllers and sensor gloves). Immersive VR environments enable researchers to study psychological phenomena that are more closely connected to the subjective experience of an individual (eg, a tall building to elicit fear) to recreate situations that elicit symptoms (eg, anxiety) or measure specific skills (eg, a kitchen to evaluate home safety). VR environments have been used to evaluate human and environmental factors associated with performing important instrumental activities of daily living (IADLs) such as driving [4], navigating public transportation [5], cooking [6], social relatedness [7], and grocery shopping [8]. The relative advantage of virtual environments over physical spaces is the ability to safely expose individuals to situations that may pose a risk in real life (eg, driving while distracted) and the ability to create controlled environments that would be extremely difficult to duplicate in a consistent, standardized fashion in real-life simulations.

### Implications of CF

CF is a common human experience that can result in serious negative consequences, such as mistakes [9,10] and accidents [11-13]. Although most healthy people experience some degree of CF at varying times, CF can become a debilitating and life-altering experience for individuals diagnosed with chronic medical conditions [14-16]. Debilitating levels of CF occur as a frequent comorbid symptom in a range of medical [17], neurological [18], and acquired conditions [19], particularly those affecting the integrity of neuronal processes [20,21]. The serious consequences of CF at work, during daily activities, and as a potential cause of disability across a broad spectrum of clinical conditions make the study of objective and subjective fatigue in healthy and clinical populations a priority across multiple disciplines.

### CF Induction

The most well-established model for inducing CF under experimental conditions is prolonged cognitive performance. Specifically, participants perform a cognitive task for an extended period (eg, 15-120 minutes) and assessments of fatigue level occur before, during, and after the fatiguing task. Various cognitive tasks reliably induce subjective feelings of fatigue, including continuously performed attention [22,23], inhibition [20,24,25], working memory [26-28], and complex cognitive activities [29-31]. Tasks requiring continuous visual monitoring for *critical events* produce a highly replicable phenomenon called the *vigilance decrement* [32], which has a moderate effect size [33]. Factors affecting the onset of vigilance decrement include image quality [34], response frequency [35], rest breaks and secondary task interruption [36], and multitasking [37]. Moderately complex cognitive functions such as working memory [27,38-41] and inhibitory control [24,42-48] tasks produce subjective feelings of fatigue but inconsistently produce performance decrements. Simple and complex vigilance tasks produce CF; however, these laboratory tasks may not best represent how CF occurs in daily life as boredom and task disengagement may account for observed vigilance decrement effects [38]. A better approach to understand CF for clinical purposes may require the evaluation and assessment of fatigue in typical daily living activities.

### Work Task and Environment Characteristics and CF

Work fatigue studies target tasks and environmental characteristics that produce CF in everyday activities. Close visual work involving inspection, comparison, or identification of details on visual images [49-53] and high rates of decision-making are sources of work fatigue [54-56]. Work interruptions interfering with workflow increase feelings of frustration [57], stress [58], and feelings of emotional exhaustion [59]. Work interruptions cause a loss of focus [60] and increase cognitive workload [61], mental effort, annoyance, frustration, and sense of time pressure [62,63]. Random, uncontrollable, interruptions in the middle of a task [61,62] that require immediate attention induce the most stress [62-64]. Individual differences in personality impact the level of perceived stress and fatigue associated with work-related tasks [63]. Work requiring intensive visual inspection or high rates of decision-making induce fatigue, and environmental factors such as distractions and interruptions significantly increase perceived frustration, workload, and fatigue.

### Daily Living and CF

Managing complex activities, such as shopping, cooking, using transportation, driving, and finances is referred to as an IADL [65]. Extensive research has focused on the relationship between driving and CF. Fatigue and cognitive workload increase with driving [66,67]. Time to fatigue while driving is hastened by extra cognitive demands, stress, distractions, multitasking, and environmental factors [66-68], although time on task and monotony are most impactful [69,70]. Personal characteristics associated with driving fatigue include fatigue proneness, dislike of driving, and coping style [66]. Surprisingly, few studies have evaluated the relationship between IADLs and CF; however, such assessments offer tremendous potential for discerning points for clinical intervention. There is some evidence that apathy, depression, and impaired cognitive functioning are risk factors for difficulties in performing IADLs [71,72]. A public transit study demonstrated that a common IADL induces cognitive workload in real life, task experience moderates perceived workload, and immersive VR provides a close approximation of the cognitive effects observed in real life [5]. The extended performance of a daily activity may induce CF, and the effects are moderated by individual and environmental factors. Grocery shopping provides an apt task for assessing CF.

### Immersive VR and Grocery Shopping

Virtual shopping environments have been used to evaluate how cognitive functions might operate in real-life situations [73-75] and may prove effective for the study of CF. Grocery shopping requires a combination of low and high levels of cognitive processes [8,73-75]. Looking for a specific product requires visual inspection, scanning, and focused attention. Traversing a shopping store requires visual attention (eg, looking for signs), spatial mapping, working memory, memory, and executive functioning [8,73-77]. Virtual shopping environments have been used successfully among individuals with significant cognitive impairment [8,78-81], and virtual shopping tasks correlate with real-life shopping activities [81,82].

We identified immersive VR grocery shopping as a suitable model to study fatigue associated with an IADL because it provides familiar but complex visual stimuli, affords the opportunity to search and choose, and presents the participant with well-known but complex cognitive challenges, such as comparisons, discernment, and decision-making. A potential disadvantage of using immersive VR to study CF is the risk of physical distress and eye strain in VR, which may confound the experience of CF or its measurement [83-85]. The risk of eye strain and other physical symptoms is reduced when high-quality head-mounted display (HMD) devices are used, motion is performed using physical walking or teleporting, the field of view is large, and each eye receives high-quality images [84]. In some cases, the realism of the environment must be sacrificed to reduce side effects.

We propose a 2-phase evaluation of the CF induction in VR. In phase 1, we will explore the feasibility of using immersive VR as a platform for studying CF using a user experience (UX) research methodology. We will use a combination of qualitative and quantitative approaches to identify components of the VR interface or environment that may contribute to feelings of eye strain or distress or make the shopping task difficult to perform. Phase 2 explores cognitive, environmental, and individual characteristics associated with VR-based grocery shopping–induced CF.

### Objectives

Despite extensive research on CF, questions remain regarding the individual and environmental characteristics that relate to CF, particularly in daily living activities. Prior studies evaluating CF in daily activities have primarily focused on driving [5,70] or very specific job-related activities [49,53,86]. We will use immersive VR to control environmental and task characteristics to identify factors that affect the onset of fatigue. Grocery shopping is used as a fatigue-induced activity because it requires multiple simple and complex cognitive functions, has been identified as a significant cause of CF in susceptible individuals [87], and is susceptible to disruption by disability [88]. On the basis of previous research, engaging healthy participants using virtual shopping environments indicates the feasibility and acceptability of VR and therefore provides the best chance of detecting the CF response [5,86,89].

In the experiment, we will replicate numerous cognitive aspects of shopping, including simultaneous and successive engagement of multiple cognitive processes including working memory, spatial planning, inhibitory control, visual search, inspection, and comparison, reading and applying information from nutritional labels, and decision-making. We can manipulate the mental workload through specific task requirements. In addition, we can test the relative effect of environmental factors, such as the effect of sound and visual cues on CF and workload, by introducing the presence of interruptions, distractions, and goal interference. In a controlled shopping environment, where interruptions can be planned carefully, as the participant executes goal-directed behaviors, real-life frustrations such as poor shelf organization and item placement, crowded conditions, noise, and other disruptions can be implemented. In future studies, the virtual shopping environment will allow us to test hypotheses related to the relationship of task difficulty, perceived task difficulty, environmental disruptions, and feelings of frustration with CF. Initial trials will use healthy controls, and subsequent studies will evaluate CF in clinical populations.

The aim of phase 1 is to evaluate the design elements of the virtual shopping environment to identify any factors that may hinder the ability of participants to effectively perform tasks in the virtual environment, identify the risk of physical distress, and obtain user feedback about realism and functionality. The primary hypothesis for the UX study is that the VR environment will be acceptable; however, some users will exhibit minor difficulties using the controllers and interacting with the environment. The primary outcome measures will be observational ratings assessing user difficulties with controller use, interacting with objects, and moving in the environment. The secondary hypotheses include that participants will report only minimal feelings of distress, will report that the virtual grocery store appears realistic and immersive, and will provide a general positive response to the experience with additional helpful ideas about how the experience could be improved.

The primary aim of phase 2 is to evaluate individual and environmental characteristics associated with susceptibility to experiencing CF in the context of performing an IADL, specifically shopping. Our primary hypothesis in phase 2 is that individuals performing structured grocery tasks will report more CF than simple exploratory behavior in the grocery store and that individuals experiencing distractions and interruptions will report more fatigue than those who do not experience interruptions. The primary outcome measure in phase 2 is the self-reported change by participants in CF by shopping experience. The secondary aims of phase 2 are to identify performance and eye-tracking measures that objectively identify fatigue, cognitive abilities, personality characteristics, shopping experience, or transient mood states that affect susceptibility to fatigue during shopping. Specific secondary exploratory hypotheses include that perceived workload increases with time on task for structured tasks and disruptive environments, percent eye closure and gaze shift increase with time on task and are associated with self-reported fatigue, and shopping accuracy declines with time on task.

## Methods

### Study Design

**Figure 1:**
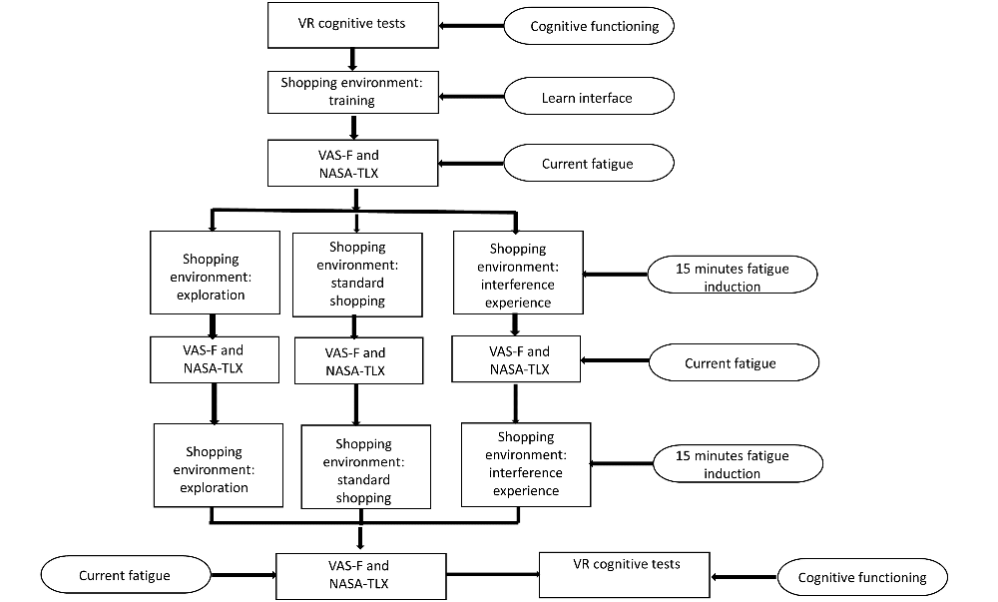
Virtual reality content in sequence. NASA-TLX: National Aeronautics and Space Administration–task load index; VAS-F: Visual Analog Scale Fatigue; VR: virtual reality.

This will be a 2-phase development (UX) and implementation (eg, randomized controlled trial) research protocol. The two phases share the same general immersive VR environment, as shown in Figure 1. The two phases diverged in the non–VR-related procedures used in each protocol. The VR sequence in each phase will follow the standard model commonly used in CF induction studies, that is, baseline cognitive assessment, baseline subjective fatigue and workload assessment, fatigue induction with a midpoint (eg, at 15 minutes) subjective assessment of fatigue, finishing with a postassessment of fatigue, and cognitive assessment. Each of these elements is shown in Figure 1.

Randomization will be used in each study to assign participants to 1 of the 3 grocery shopping experiences: shopping exploration, standard shopping, and shopping interference. In each study phase, participants completed a brief self-reported medical history to rule out conditions associated with chronic fatigue, cognitive impairment, or susceptibility to seizures. Participants in both studies completed the Virtual Reality Symptom Questionnaire (VRSQ) [90] before VR immersion and immediately after VR immersion. These procedures will help differentiate the impact of VR immersion from the fatigue induced by the shopping task.

**Figure 2:**
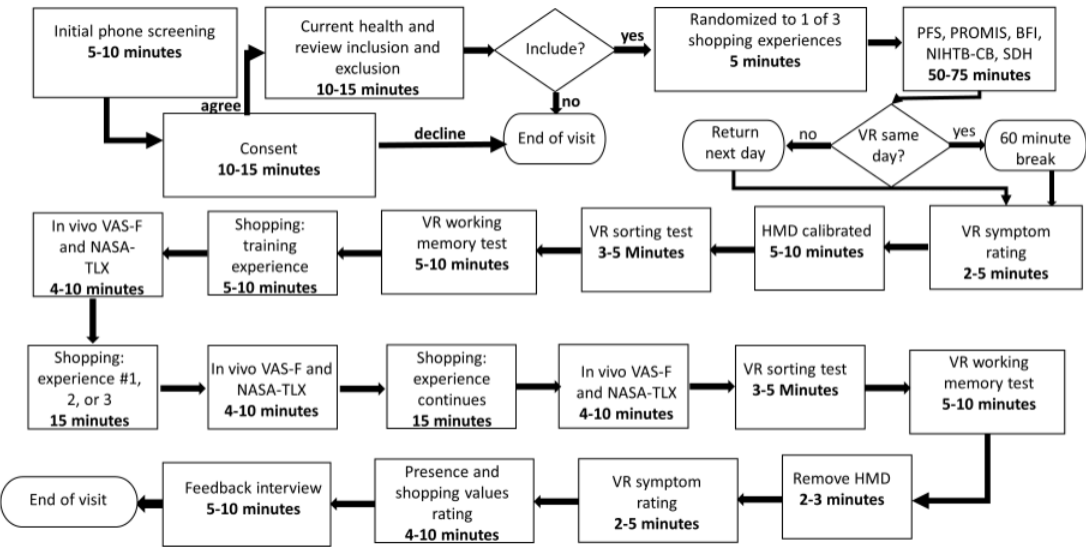
Detailed procedure of the cognitive fatigue study. BFI: Big Five Inventory; HMD: head-mounted display; NASA-TLX: National Aeronautics and Space Administration–task load index; NIHTB-CB: National Institutes of Health toolbox–cognition battery; PFS: Pittsburgh Fatigability Scale; PROMIS: Patient-Reported Outcomes Measurement Information System; SDH: social determinants of health; VAS-F: Visual Analog Scale Fatigue; VR: virtual reality.

The phase 1 study will evaluate the participants’ capacity to learn to interact with objects in the virtual environment, navigate within the grocery store environment, read and respond to information and questionnaires, and identify any early adverse effects of VR exposure. The data collected from this study will be used to improve the VR interface and modify the participant interactions or the length of exposure. The study staff will observe the engagement of the participant in the immersive task by viewing the person as well as by viewing their exact point of view on a separate computer screen. Participants will complete rating scales including feelings of presence [91] in the shopping environment, self-reported simulator sickness symptoms [90], and shopping values or experience [92]. All participants completed a standardized UX interview. The phase 2 study protocol, detailed in Figure 2, will incorporate additional self-report and performance measures (see Table 1 for lists of measures in each phase). Additional measures include state and trait measures of fatigue [93,94], current emotional state (ie, anxiety and depression) [94], personality traits [95], and cognitive functioning [96]. These measures will be completed before the VR portion of the study with a 1-hour break between completing additional study measures and VR immersion. Similar to phase 1, participants will complete measures of presence [91] and shopping values or experience [92] to assess the impact of realism, shopping as a pleasant versus utilitarian task, and frequency of grocery shopping in real life on fatigue and performance. A brief post-VR interview will be completed to obtain additional insight about the environment and to debrief participants about the purpose of the study.

**Table 1:** Assessments by phase.

Assessments	Phase 1	Phase 2
Virtual Reality Symptom Questionnaire	✓^a^	✓
Immersive VR^b^ cognitive tests	✓	✓
Immersive VR Visual Analog Scale–Fatigue	✓	✓
Immersive VR NASA-TLX^c^	✓	✓
Presence Questionnaire	✓	✓
Shopping Values Questionnaire	✓	✓
Social Determinants of Health	✓	✓
User Experience Interview	✓	
PROMIS^d^ Depression		✓
PROMIS Anxiety		✓
PROMIS Fatigue		✓
Pittsburgh Fatigability Scale		✓
Big Five Inventory		✓
National Institutes of Health Toolbox–Cognition Battery		✓

^a^Assessment is present.

^b^VR: virtual reality.

^c^NASA-TLX: National Aeronautics and Space Administration–task load index.

^d^PROMIS: Patient-Reported Outcomes Measurement Information System.

### Kitchen Tasks

The participants will be seated while performing the tasks in the kitchen environment. The participant will appear to be seated in a kitchen table with a pillbox and pill bottles in front of them. In the first task, the participant will be instructed to correctly select the pillbox compartment (labeled with the days of the week) where each pill belongs. A calendar on the table shows an image of each pill and the pillbox location (eg, Sunday or Monday); when a pill appears in front of the examinee, they will select the correct pillbox location by using a scroll and trigger pull sequence. An animation sequence will show the pill entering the selected location. Another pill will appear with a sound alert until 120 seconds have passed or 120 pills have been sorted.

In the working memory task, the participant will be shown a series of pills by day and time of day associations. Using the same calendar concept, the participant will see where 2 pills are to be placed in the pillbox (eg, red pill in the morning on Monday and blue pill in the evening on Friday) for 10 seconds. They were instructed to remember the location of each pill. The key will be taken out of view and the pills will appear one at a time. Each participant will select the location where each pill belongs using a scroll and trigger sequence. The task will increase in difficulty with 3, 4, 5, and 6, the number of pill locations to recall. The task will end when the participant obtains four consecutive scores of zero.

### Shopping Tasks and Experiences

**Figure 3:**
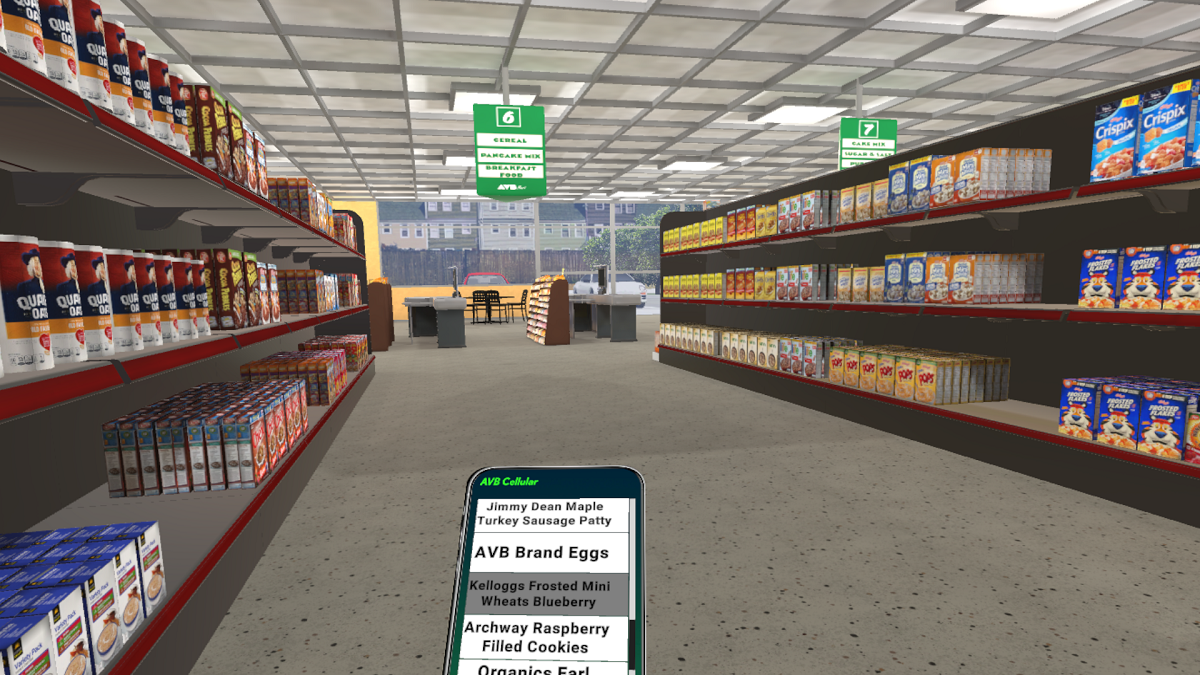
Grocery store screenshot.

Participants will remain seated during all VR shopping experiences. Product labels are legible for brands and specific products without selecting the object. Product selection enables the viewing of all product details. Products will be selected off a shelf using a wand controller acting as a pointing device (eg, laser beam), followed by a point, highlight, and trigger pull selection sequence. Selected product labels will appear on a virtual cell phone in front of the participant with a menu of options (eg, buy product, return product, and review shopping list). Participants traverse the store using a restricted teleport feature. Movement will be restricted to a more realistic experience and to avoid long-distance movements that might result in disorientation and difficulties in learning the store layout. Figure 3 presents a screenshot of the grocery store and shows the cell phone, products, and aisle.

### Shopping Training

The participant will appear in a small version of the grocery store. They will be instructed on how to use the virtual cell phone to check their shopping list, review items in their cart, and answer text messages with the left-hand controller. They will be instructed on how to teleport and select items off the shelf with the right-hand controller. In the shopping training task, the participant must follow specific directions and must correctly put four items in their cart from the shopping list, one of which must be returned to the shelf, and they must answer a text to complete the shopping training. To complete the shopping portion of the task, the participant will need to teleport successfully to multiple shelves and aisles.

### Shopping Experience Number 1

Experience number 1 will be a control experience that will allow the participant to explore the grocery store with no specific task to complete. The shopping environment includes a few avatars and some low music in the background to simulate a realistic shopping environment during off-hours. All shopping actions will be enabled, and the participant may select items and place them in the shopping cart. The only requirement will be that they remain in the environment for 30 minutes. The control experience will evaluate whether the VR environment itself induces significant fatigue that may confound the interpretation of task-specific fatigue induction.

### Shopping Experience Number 2

Experience number 2 is a standard shopping experience designed to mimic a realistic shopping experience during a typical day. Participants will be provided with a shopping scenario. They will be told that they are shopping for sick friends. The participant will try to obtain as many items as possible from the cell phone shopping list. Participants will traverse the grocery store to find objects on the list and place them in the shopping cart. Avatars are present in the store but do not hinder progress or create any specific distractions. The background sound includes typical background noise, music, and overhead announcements. This condition assesses the cognitive load and fatigue related to the mental activity of shopping.

### Shopping Experience Number 3

Experience number 3 will be the standard shopping experience with frustrating and interrupting events. Participants will be provided the same shopping scenario as experience number 2; however, this shopping experience will be designed to mimic very high traffic, a holiday shopping experience, store crowding, misplaced items, and loud distractions. In addition to environmental stressors, the cell phone will receive *texts* from the friend requesting changes to the grocery list after items have already been selected. Text alerts will be short, repetitive, high-pitched sounds that continue until the text is answered. The progress of the participants will be impeded by an avatar standing in front of a needed item, an aisle blocked for a spill, or a palette blocking access to a specific shelf area. The sounds of a baby crying, people talking, coughing, laughing, and sneezing are present. The music and announcements are played at a slightly higher volume than in the standard shopping condition. This condition will assess the cognitive load and fatigue related to the mental activity of shopping in the presence of distractions and frustrating events.

### Fatigue Assessment

Fatigue induction studies evaluate real-time changes in fatigue symptoms by self-reporting, performance, and eye tracking. An adapted version of the Visual Analog Scale–Fatigue (VAS-F) [97] will be used as a state fatigue measure given its history of use in fatigue induction research. [27,98,99] A closely linked concept to CF is cognitive workload. Cognitive workload applies an ergonomic and human factors model (eg, elements of a job or task that create a feeling of mental work) to understand fatigue as it relates to sustained work performance [100-104]. The NASA-TLX is a commonly used measure of workload [105,106]. In addition to subjective measures, there are two approaches to use performance data to objectively measure fatigue: change in performance on the induction task or using a pre- versus postintervention cognitive assessment [26,29,107,108]. Tests of reaction time [42], working memory [23], and inhibitory control [32] are used to assess fatigue effects.

Psychophysiological measures identify objective brain or autonomic nervous system indicators of fatigue using EEG (electroencephalogram) [22,23,30,109], ERP (event related potential) [28,31,86,110], functional brain imaging [27,40,107,108], and ECG (electrocardiogram) [29,30] to measure changes in brain or cardiovascular activity associated with fatigue. Of the various physiological indicators, eye tracking has emerged as a promising, noninvasive tool for identifying objective measures of CF. Eye tracking studies show changes in blink rate, percent eye closure, gaze fixation (eg, length and location), and gaze shift rate are associated with CF [30,49-51,111-114]. Changes in gaze shift rate may indicate use of less efficient lower-level cognitive processing [49] and a centralized fixation can indicate a loss of full attention to the task [115]. Several sources, using different task demands, show changes in visual activity as the time on task increases.

### Engineering and Technology

The virtual environment was created using Unity 3D (Unity Technologies). Products will be created by the graphics design team using digital image files obtained from the product manufacturer, labels scanned from acquired grocery items, or modified from items purchased through the Unity Asset Store. All labels are converted into 3D objects using a variety of programs and techniques. The design team will develop a cohesive store branding and coordinated color scheme for store assets. Within the environment, near objects will be displayed with a high degree of visual detail, whereas distant objects will have reduced detail. General product labeling will be legible without selecting the object; however, specific product information (eg, reduced sodium or nutritional values) will only be legible after product selection.

The VIVE Pro Eye (HTC Corporation) will be the HMD device used in each study. This device has a 2880 × 1600-pixel display resolution and includes eye tracking and high-resolution surround sound and allows for the use of glasses and adjustable optics that are designed to minimize eye fatigue and cybersickness. Participants will interact with the virtual environment and objects within the environment by using a wand. The VR program is delivered to the HMD via a display port from a Dell Precision workstation 7920. The technical features of the 7290 include Intel Xeon Gold 5122 3.6 GHz, 3.7 GHz Turbo, 4C, 10.4 2UPI, 16.5 MB Cache, NVIDIA Quadro P5000, 16 GB, 4 DP, and 32 GB 2 × 16 GB DDR4 2666 MHz RDIMM ECC (error correction code) memory. This equipment will have adequate processing power, graphical speed and resolution, and memory to provide a vivid, smooth immersive experience. Eye-tracking data will be collected from individual participants using the integrated eye-tracking system contained within the HTC Vive Pro HMD. The data sampled by the HMD eye tracker include data output (eye information): timestamp (device and system), gaze origin, gaze direction, pupil position, pupil size, and eye openness, which are captured every 200 ms.

### Participants

The participants will be recruited from a local metro region. We anticipate that participant background characteristics (eg, education, ethnicity, sex, and age) will be representative of the metro area in background characteristics (eg, education, ethnicity, sex, and age). Recruitment will be managed by the National Institutes of Health (NIH) Office of Patient Recruitment, using local flyers; Office of Patient Recruitment website; and posts on social media, including Facebook and Twitter. Participants will be remunerated to participate in the study. All protocol activities will take place in a local NIH facility in Bethesda, Maryland. 

For each phase of the study, participants will be healthy individuals aged 18-75 years. Recruitment for phase 1 will target an older (≥55 years) and younger group (18-54 years) with 50% targeted for each group, stratified by sex. Recruiting a broad age range will ensure usability among older individuals, as future apps will likely involve older adult clinical populations. The sample will be stratified by sex, as some research suggests that women may experience immersive VR differently from men [116,117]. The phase 1 sample size will be 24, with 8 participants completing each of the three shopping conditions. Evaluating participants from a variety of backgrounds is important in UX research to identify any systematic issues in the interface, content, or instructions. The phase 2 study recruited 60 healthy individuals aged 18-75 years. For this study, there will be no targeted recruitment of older adults, as any design issues specifically associated with subject age will be addressed before phase 2. The sample size was determined based on the calculated effect sizes of fatigue induction studies that used the VAS-F (Cohen *d*=0.65; SD 0.25) and vigilance decrement studies [34]. The inclusion and exclusion criteria are included in Textbox 1.

**Inclusion and exclusion criteria.**

**Inclusion criteria**

- Participants aged 18-75 years
- Willingness to complete the study procedure
- Willing to provide feedback on virtual reality experience
- Able to provide consent

**Exclusion criteria**

- Self-reported
  - Any impairment in visual functioning (eg, 3D depth perception, color blindness, visual acuity, and oculomotor control) not corrected with lenses
  - Eye pain or iritis
    Susceptibility to photosensitive seizures or diagnosis of seizure disorder
  - Inability to use hands
    Diagnosis of neurological conditions
  - Diagnosis of sleep disorders
  - Current treatment for chronic physical pain, migraines, any diagnosis of a clinical condition associated with cognitive or physical fatigue (eg, multiple sclerosis and chronic fatigue syndrome)
  - History of acquired brain injuries
    Current cold or flu symptoms
- National Institute of Nursing Research employees and staff or subordinates, relatives, and coworkers of National Institute of Nursing Research employees and staff or a study investigator
- Not fluent in English
- For phase 2, participation in phase 1

### Analysis

#### Overview

This statistical analysis plan was reviewed by the National Institute of Nursing Research (NINR) statistician. All data will be processed, cleaned, and analyzed using the SAS 9.4 (SAS Institute). The data analysis approach for phase 1 focuses on descriptive and nonparametric tests. The primary goal of the phase 1 study is to evaluate the measures and identify any interface issues that cause participants to have problems interacting with the environment or producing unexpected physical symptoms. The phase 2 study will test specific hypotheses using inferential statistics.

#### Phase 1

The data analysis for phase 1 will inform decisions related to programming, data outputs, adequacy of obtained score distributions, evaluation of the psychometric quality of the cognitive tests, and identification of any potential confounds (eg, length of VR exposure) that could impact future studies. We will examine the initial evidence for the fatigue induction effects of the three shopping conditions. We will use frequency and nonparametric procedures to evaluate the rates of observed difficulties using controllers, interacting with the environment, and following instructions generally, by age groups and by sex. We will compare self-reported feelings of distress before entering the VR environment to the self-reported symptoms after exiting the VR environment. For this analysis, the Wilcoxon signed-rank test will be used. Secondary analyses evaluate distributions of key dependent measures including self-reported CF and workload, eye-tracking data (eg, blink rate, percent eye closure, gaze fixation length, and gaze shifts), and performance data for shopping and cognitive tasks (eg, correct response and response speed), as having a score distribution of several SDs will be important when the measures are applied in hypothesis testing. Following a structured interview, responses will be analyzed for common interface or immersive content issues (eg, difficulty teleporting, difficulty reading text, and problems accessing grocery list).

For example, in phase 1, we will compare the participants’ self-reported physical symptoms and eye-related symptoms from the VRSQ before versus after completing the VR shopping experience. We will use the Wilcoxon signed-rank test, given the high probability of a nonnormal distribution in the dependent measure. This comparison will provide evidence to determine whether the VR environment produces physical distress or eyestrain. We computed the total scores for each of the observation scales. These totals inform about the number of times the participant had difficulties with the interface. We will compare the frequencies of interface problems in older and younger and male and female subjects using the chi-square test. These are structured statistical analyses planned as part of the formal UX results. Exploratory procedures are used to identify the relationship between user behavior (eg, number of items reviewed, distance traveled in the environment, and accuracy of shopping behaviors with measures of shopping enjoyment, shopping experience, and sense of immersion in the environment). For these analyses, we used Spearman rank-order correlations.

#### Phase 2

We will use the repeated measures analysis of variance (ANOVA) with the VAS-F and NASA-TLX as repeated dependent variables by shopping experience (fixed) to test the hypothesis that grocery shopping creates fatigue and workload, particularly when the person experiences interruptions and distractions. Secondary analyses will evaluate whether objective indicators of fatigue, such as eye tracking, shopping performance, and cognitive functioning (eg, pre- and postshopping processing speed and working memory) significantly differ by shopping experience using repeated measures ANOVA. A third series of analyses evaluated the relationship among individual characteristics, perceived CF, and workload. We will primarily use correlation to evaluate the relationship among pre-existing symptoms of fatigue, anxiety depression, personality traits, and fatigue susceptibility. Cognitive measures from the NIH toolbox will be correlated with perceived fatigue and workload to identify whether cognitive abilities influence the perception of cognitive workload and fatigue.

For example, we will use the repeated measures ANOVA to test the primary hypothesis that the cognitive activity of shopping for specific items will create a greater perception of mental workload and fatigue compared with just exploring the environment unless the distribution of dependent measures does not allow for using this specific statistical procedure. Similarly, we will use an appropriate correlation procedure to compare the level of activity measures such as distance traversed in the store, number of items selected and reviewed, and efficiency and accuracy of shopping activity with perceptions of fatigue and workload. Correlation procedures will be used to assess the relationships between constructs, such as personality style, cognitive ability, fatigue susceptibility with self-reported mental workload, and fatigue to identify individual differences in fatigue susceptibility. Eye tracking such as percent eye closure will be explored as a possible objective indicator of fatigue by serving as a dependent measure in the repeated measures ANOVA by shopping experience and in correlational analysis with self-reported fatigue and workload. The actual analysis considers the appropriateness for each specific variable distribution.

## Results

This study was approved by the scientific review committee of the NINR and identified as an exempt study by the institutional review board of the NIH. Data collection will begin in spring 2021.

## Discussion

### Overview

The development of a complex, immersive VR environment requires close collaboration between individuals from multiple disciplines. The iterative design of the grocery store involves simulation of activities (eg, selecting objects using various techniques), legibility assessment of various product creation strategies, user testing by team members to identify potential sources of physical discomfort (eg, effect of antialiasing on visual acuity and developing headache), comparison of movement modalities (eg, walking vs sitting), ambient environmental factors (eg, store sounds and signage), and sizing of store elements (eg, shelf height, length, and store size). In addition, the research team will implement several simulations to evaluate the software performance and integrity of the data outputs. For each activity, the team of engineers, graphic designers, clinical experts, and researchers evaluated the relative impact of design on study requirements, UX, and software functionality. This process requires a high degree of communication and knowledge sharing.

The digital development process is fraught with potential pitfalls, particularly if team communication breaks down, and a collaborative spirit is diminished. For example, the design of the user interface can have a significant effect on the cognitive demands of using the software. If not created collaboratively, the resulting user interface may create a confound in the interpretation of the cognitive processes required for performing an IADL, as unintended skills may be introduced into the process. When communication is effective, multiple options for the experience are evaluated, such as comparing the use of different processes to remove an individual item from a shelf. Some of these options produce unintended consequences associated with product legibility and the potential for users to develop headaches from the experience. However, a seemingly less natural object selection process (eg, point and trigger pull) alleviates these issues with only a slight reduction in the sense of realism. Similarly, creating intricately detailed products had a negative effect on software functionality (eg, lower flicker fusion rate), which produces an unpleasant experience for the user. By reducing the object vectors and polygons, it is possible to maintain a high degree of realism without interfering with the software functionality. Researchers wishing to deploy complex, immersive VR experiences must anticipate the myriad of factors that potentially introduce confounding variance that reduces the fidelity of an intervention or the measurement of key constructs. In our experience, team communication of design requirements, relying on an interdisciplinary set of skills and knowledge, continuous informal UX testing, and applying an iterative design approach are necessary for effectively using VR as a research platform.

### Strengths and Limitations of This Study

CF is a complex phenomenon influenced by task, environment, personal experience, and individual differences. Our experimental conditions included a familiar task performed in a realistic immersive VR environment that allows for the precise control of stimuli. The ability to control stimuli and timing of events will enable us to determine the relative contribution of distraction, boredom, task complexity, and person characteristics on the development of CF. The strength of the immersive VR experience is the capacity to create a cognitive experience that closely aligns with real-life demands. Our ability to control the presence and timing of interfering factors enables us to assess environmental influences that would be almost impossible to standardize using an actual grocery store.

The immersive VR environment allows us to seamlessly use multiple measures of CF. We will use subjective indicators of CF and workload to better understand how perceived fatigue (eg, physical fatigue: *tiredness* and *sleepiness;* cognitive fatigue: *efficiency* and *difficulty in concentrating*) and workload (eg, mental demand, effort, and frustration) relate to the effects of tasks, environments, and other factors. Potential objective measures of CF, such as changes in behavior (eg, performance efficiency, shopping list rechecking, rate, and the efficiency of movement) and changes in eye movement, can be measured unobtrusively. The use of a randomized controlled design is a strength of this study. Participants will be randomly assigned to 1 of the 3 shopping conditions to control for any confounding effects of person-level background characteristics (eg, age) that may affect fatigue or reactions to the VR experience.

The primary weakness of the study is the potential for the immersive VR environment itself to create feelings of eye strain and fatigue. This visual effect of the VR environment may have a stronger impact compared with the fatigue effects of the cognitive task, reducing the observed differences between experiences. We are mitigating the potential of VR-induced fatigue by using high-resolution HMDs. In addition, we will measure symptoms of physical distress pre- and postimmersion to identify any signs of physical distress that could affect the levels of self-reported fatigue. We are limiting the potential for motion sickness by using the teleport function for movement and other changes to the visual presentation to minimize any potential for headaches. The UX study is performed to specifically address questions of usability, including identifying any factors that might produce physical discomfort.

### Conclusions

Our initial informal user testing indicated a high sense of immersion and realism in the virtual shopping experience. We will continue to modify the shopping experience to meet the research goals of evaluating the effect of cognitive and emotional factors that influence fatigue onset. The store size will be 18,000 square feet, consistent with the dimensions of a small grocery store in the United States with hundreds of unique items created. Additional products are being created to give the store correct proportionality, typicality in selection options, and a visual experience that is consistent with a grocery shopping experience in the United States. The software will be ready for formal UX testing as outlined in this paper in the spring of 2021. We anticipate that the virtual shopping experience will provide a wealth of data related to the experience of CF while performing routine activities.

## Abbreviations

ANOVA: analysis of variance
CF: cognitive fatigue
ECC: error correction code
HMD: head-mounted display
IADL: instrumental activity of daily living
NIH: National Institutes of Health
NINR: National Institute of Nursing Research
UX: user experience
VAS-F: Visual Analog Scale–Fatigue
VR: virtual reality
VRSQ: Virtual Reality Symptom Questionnaire
